# The genome sequence of a snail-killing fly, *Pherbina coryleti* (Scopoli, 1763)

**DOI:** 10.12688/wellcomeopenres.18790.1

**Published:** 2023-01-19

**Authors:** Olga Sivell, Ryan Mitchell, Duncan Sivell, Natural History Museum Genome Acquisition Lab, Darwin Tree of Life Barcoding collective, Wellcome Sanger Institute Tree of Life programme, Wellcome Sanger Institute Scientific Operations: DNA Pipelines collective, Tree of Life Core Informatics collective, Darwin Tree of Life Consortium

**Affiliations:** 1 Natural History Museum, London, UK

**Keywords:** Pherbina coryleti, snail-killing fly, genome sequence, chromosomal, Diptera

## Abstract

We present a genome assembly from an individual male *Pherbina coryleti* (snail-killing fly; Arthropoda; Insecta; Diptera; Sciomyzidae). The genome sequence is 863 megabases in span. Most of the assembly is scaffolded into six chromosomal pseudomolecules, including the assembled X sex chromosome. The mitochondrial genome has also been assembled and is 20.9 kilobases in length. Gene annotation of this assembly on Ensembl identified 32,619 protein coding genes.

## Species taxonomy

Eukaryota; Metazoa; Ecdysozoa; Arthropoda; Hexapoda; Insecta; Pterygota; Neoptera; Endopterygota; Diptera; Brachycera; Muscomorpha; Sciomyzoidea; Sciomyzidae; *Pherbina*; *Pherbina coryleti* (Scopoli, 1763) (NCBI:txid1096077).

## Background

*Pherbina coryleti* is a snail-killing fly from family Sciomyzidae (Diptera), tribe Tetanocerini. It is a yellowish, medium-sized fly (8.6–9.3 mm) with infuscated, distinctly patterned wings. It closely resembles *Pherbina intermedia* (northern European species, not recorded in Britain). Several features allow for their separation, such as strong setae on the mesopleuron (three in *P. coryleti*, one in *P. intermedia*), the shape of antennae (stout and with convex upper and lower margins in *P. coryleti*, slender and with parallel upper and lower margins in *P. intermedia*) and the patterning of cell cu ( Rozkošný, 1984; Rozkošný, 1987). Larvae and pupae were described by Knutson, Rozkošný and Berg (1975), and keys allowing for their differentiation from *P. intermedia* were given by ( Rozkošný, 2002).

*Pherbina coryleti* is univoltine (with one generation per year). Mating occurs in spring/early summer and oviposition is delayed for several months ( Knutson & Vala, 2011). The eggs are laid in batches, on plant stems or leaves in moist conditions ( Beaver, 1973). The larvae are semi-aquatic, but weak swimmers. They are predators and saprophages of a wide range of non-operculate (mainly freshwater) snails encountered on moist and exposed surfaces ( *e.g.*, stranded). The larva ruptures the haemocoel of a freshwater snail and feeds on its flesh and occasionally on haemolymph. The snail dies within minutes. Each larva consumes between 10–20 snails in its lifetime ( Knutson & Vala, 2011). *Pherbina coryleti* is a wasteful feeder, killing many more snails than needed for its development ( Beaver, 1974). The species overwinters as a third instar larva. The fly pupates away from the host. Floating puparia and mature larvae may be found in spring amongst marginal vegetation ( Rozkošný, 1984; Speight & Knutson, 2012).

This Eurasian species is common and widely distributed in Britain. It can be found in wetland habitats such as inland lakes, marshes (including coastal), fens, seasonally-flooded unimproved grassland, reeds and tall sedge beds. The flight period is from May to September ( Ball, 2017; Speight & Knutson, 2012).

The monophyly of the tribe Tetanocerini (Sciomyzidae, Sciomyzinae) is well supported by morphological ( Barker *et al.*, 2004; Marinoni & Mathis, 2000) and phylogenetic studies ( Tóthová *et al.*, 2013). Tóthová *et al.* (2013) placed *Pherbina*, *Limnia* and *Trypetoptera* in a single clade, while a previous morphological study ( Barker *et al.*, 2004) indicated the close relationship between *Pherbina* and *Trypetoptera*, *Dictyodes* and *Ilione*. More research is needed to resolve relationships between the genera within Tetanocerini.

The high-quality genome sequence described here is the first one reported for *P. coryleti* and has been generated as part of the Darwin Tree of Life project. It will aid in the study of the species as well as the evolution and phylogenetics of the group.

### Genome sequence report

**Figure 1.**
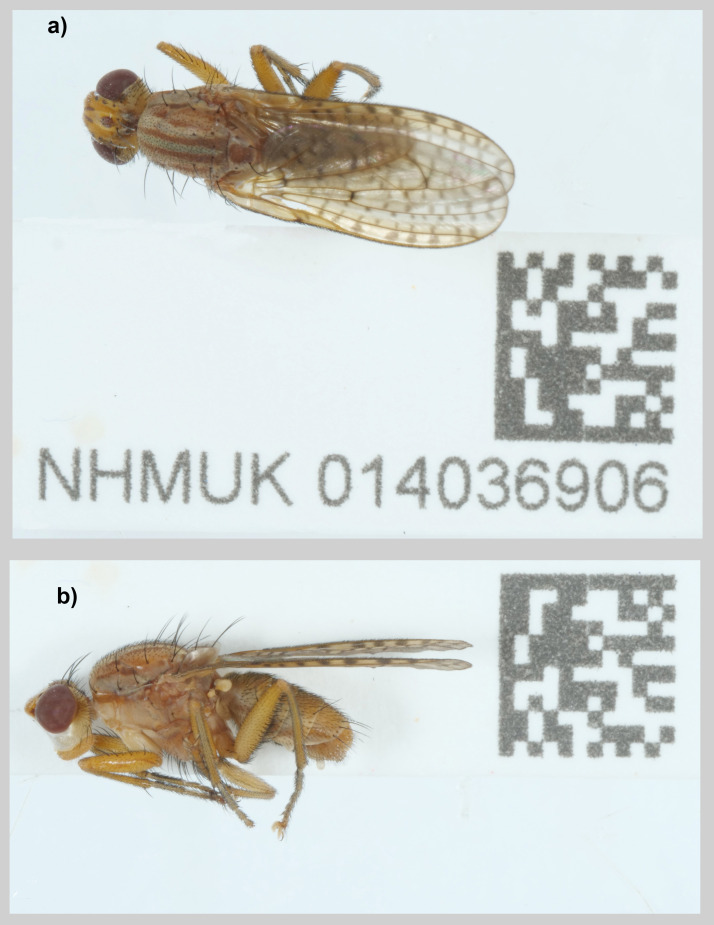
Photographs of the *Pherbina coryleti* (idPheCory1) specimen used for genome sequencing. **a**) A male habitus in dorsal view, **b**) A male habitus in lateral view. Photographs by Olga Sivell.

The genome was sequenced from one male *P. coryleti* specimen ( Figure 1) collected from Parsonage Moor, UK (latitude 51.69, longitude –1.33). A total of 23-fold coverage in Pacific Biosciences single-molecule HiFi long reads was generated. Primary assembly contigs were scaffolded with chromosome conformation Hi-C data. Manual assembly curation corrected 417 missing joins or mis-joins and removed 13 haplotypic duplications, reducing the assembly length by 5.83% and the scaffold number by 92.41%, and increasing the scaffold N50 by 3.25%.

**Figure 2.**
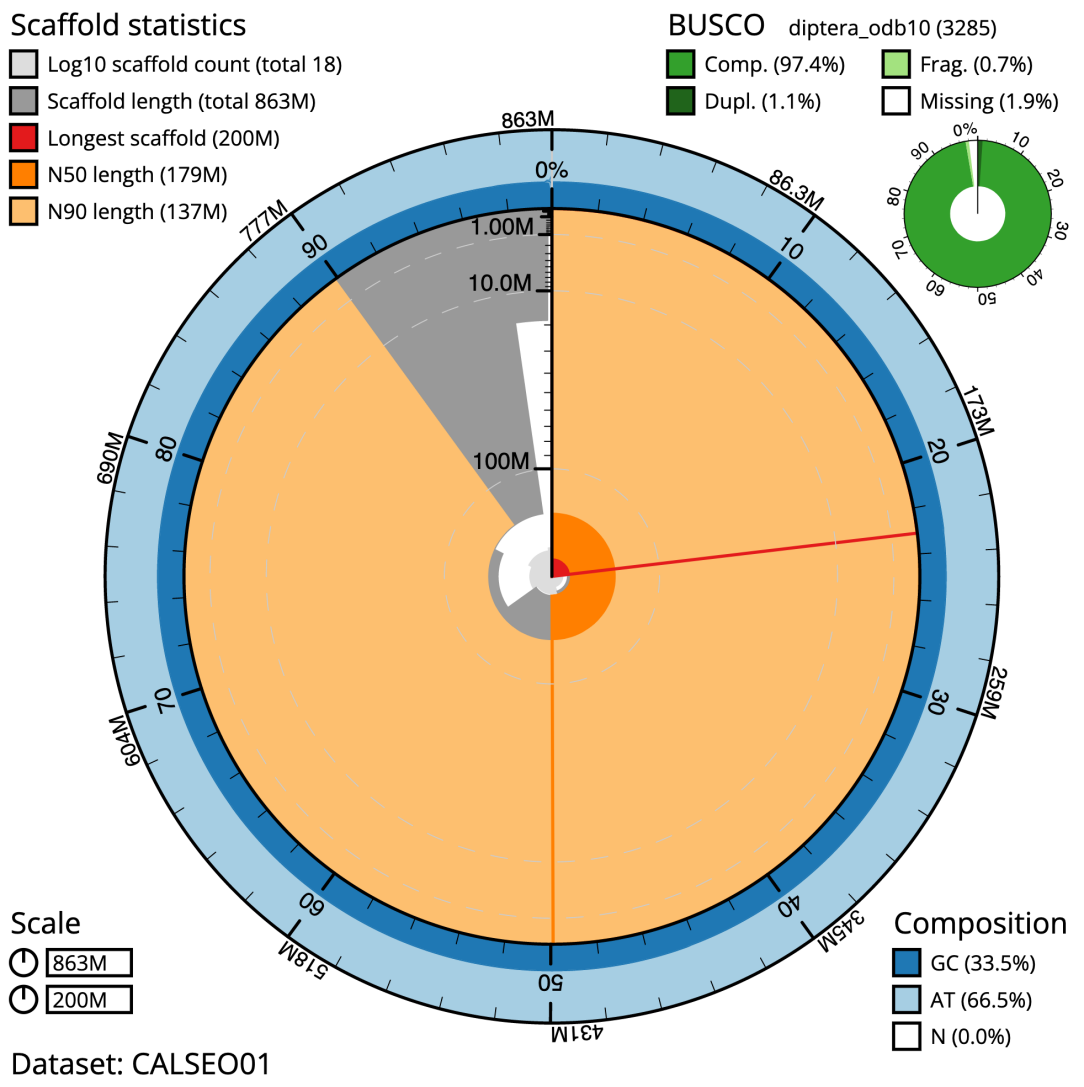
Genome assembly of *Pherbina coryleti*, idPheCory1.1: metrics. The BlobToolKit Snailplot shows N50 metrics and BUSCO gene completeness. The main plot is divided into 1,000 size-ordered bins around the circumference with each bin representing 0.1% of the 862,972,526 bp assembly. The distribution of scaffold lengths is shown in dark grey with the plot radius scaled to the longest sequence present in the assembly (199,727,623 bp, shown in red). Orange and pale-orange arcs show the N50 and N90 scaffold lengths (178,934,347 and 136,521,607 bp), respectively. The pale grey spiral shows the cumulative scaffold count on a log scale with white scale lines showing successive orders of magnitude. The blue and pale-blue area around the outside of the plot shows the distribution of GC, AT and N percentages in the same bins as the inner plot. A summary of complete, fragmented, duplicated and missing BUSCO genes in the diptera_odb10 set is shown in the top right. An interactive version of this figure is available at https://blobtoolkit.genomehubs.org/view/idPheCory1.1/dataset/CALSEO01/snail.

The final assembly has a total length of 863.0 Mb in 17 sequence scaffolds with a scaffold N50 of 178.9 Mb ( Table 1). Most (99.79%) of the assembly sequence was assigned to six chromosomal-level scaffolds, representing five autosomes and the X sex chromosome. Chromosome-scale scaffolds confirmed by the Hi-C data are named in order of size. Scaffolds 72, 42, 127, 59, 153, 102, 159, 29 are unlocalised scaffolds. One or more of these could be the Y chromosome, or could belong to the X chromosome ( Figure 2– Figure 5; Table 2). The assembly has a BUSCO v5.3.2 ( Manni *et al.*, 2021) completeness of 97.4% (single 96.3%, duplicated 1.1%) using the OrthoDB v10 diptera reference set ( *n* = 3,285). While not fully phased, the assembly deposited is of one haplotype. Contigs corresponding to the second haplotype have also been deposited.

**Table 1.** Genome data for *Pherbina coryleti*, idPheCory1.1.

Project accession data		
Assembly identifier	idPheCory1.1	
Species	*Pherbina coryleti*	
Specimen	idPheCory1	
NCBI taxonomy ID	1096077	
BioProject	PRJEB52655	
BioSample ID	SAMEA11025047	
Isolate information	male, idPheCory1 (PacBio and Hi-C)	
Assembly metrics *		*Benchmark*
Consensus quality (QV)	58.3	*≥ 50*
*k*-mer completeness	100%	*≥ 95%*
BUSCO **	C:97.4%[S:96.3%,D:1.1%], F:0.7%,M:1.9%,n:3,285	*C ≥ 95%*
Percentage of assembly mapped to chromosomes	99.79%	*≥ 95%*
Sex chromosomes	X chromosome assembled	*localised homologous pairs*
Organelles	Mitochondrial genome assembled	*complete single alleles*
Raw data accessions		
PacificBiosciences SEQUEL II	ERR9793192	
Hi-C Illumina	ERR9710922	
Genome assembly		
Assembly accession	GCA_943735915.1	
*Accession of alternate haplotype*	GCA_943735905.1	
Span (Mb)	863.0	
Number of contigs	576	
Contig N50 length (Mb)	3.3	
Number of scaffolds	17	
Scaffold N50 length (Mb)	178.9	
Longest scaffold (Mb)	200.0	
**Genome annotation**		
Number of protein-coding genes	32,619	
Gene transcripts	33,571	

* Assembly metric benchmarks are adapted from column VGP-2020 of “Table 1: Proposed standards and metrics for defining genome assembly quality” from ( Rhie *et al.*, 2021). ** BUSCO scores based on the diptera_odb10 BUSCO set using v5.3.2. C = complete [S = single copy, D = duplicated], F = fragmented, M = missing, n = number of orthologues in comparison. A full set of BUSCO scores is available at https://blobtoolkit.genomehubs.org/view/idPheCory1.1/dataset/CALSEO01/busco.

**Figure 3.**
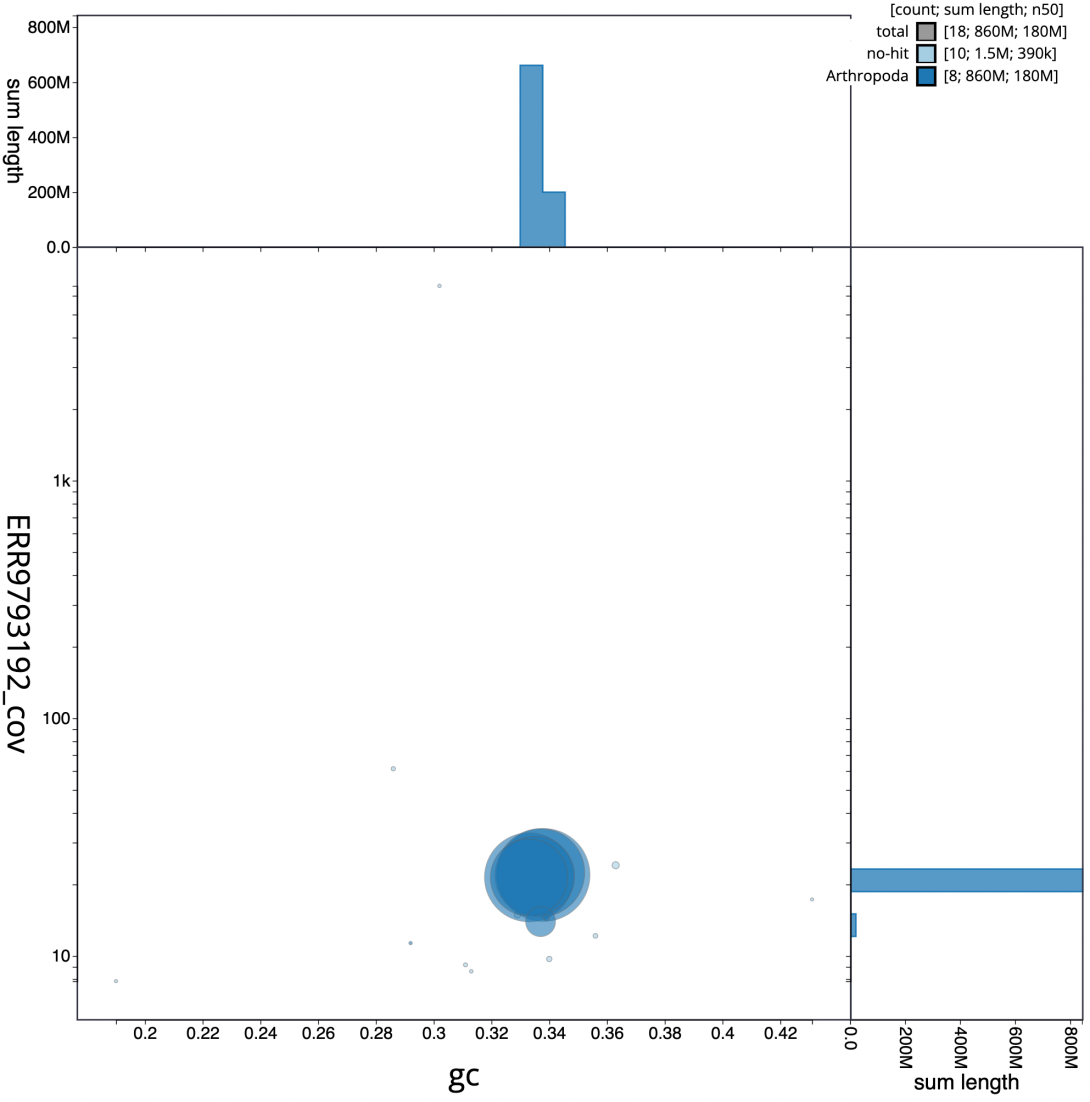
Genome assembly of *Pherbina coryleti*, idPheCory1.1: GC coverage. BlobToolKit GC-coverage plot. Scaffolds are coloured by phylum. Circles are sized in proportion to scaffold length. Histograms show the distribution of scaffold length sum along each axis. An interactive version of this figure is available at https://blobtoolkit.genomehubs.org/view/idPheCory1.1/dataset/CALSEO01/blob.

**Figure 4.**
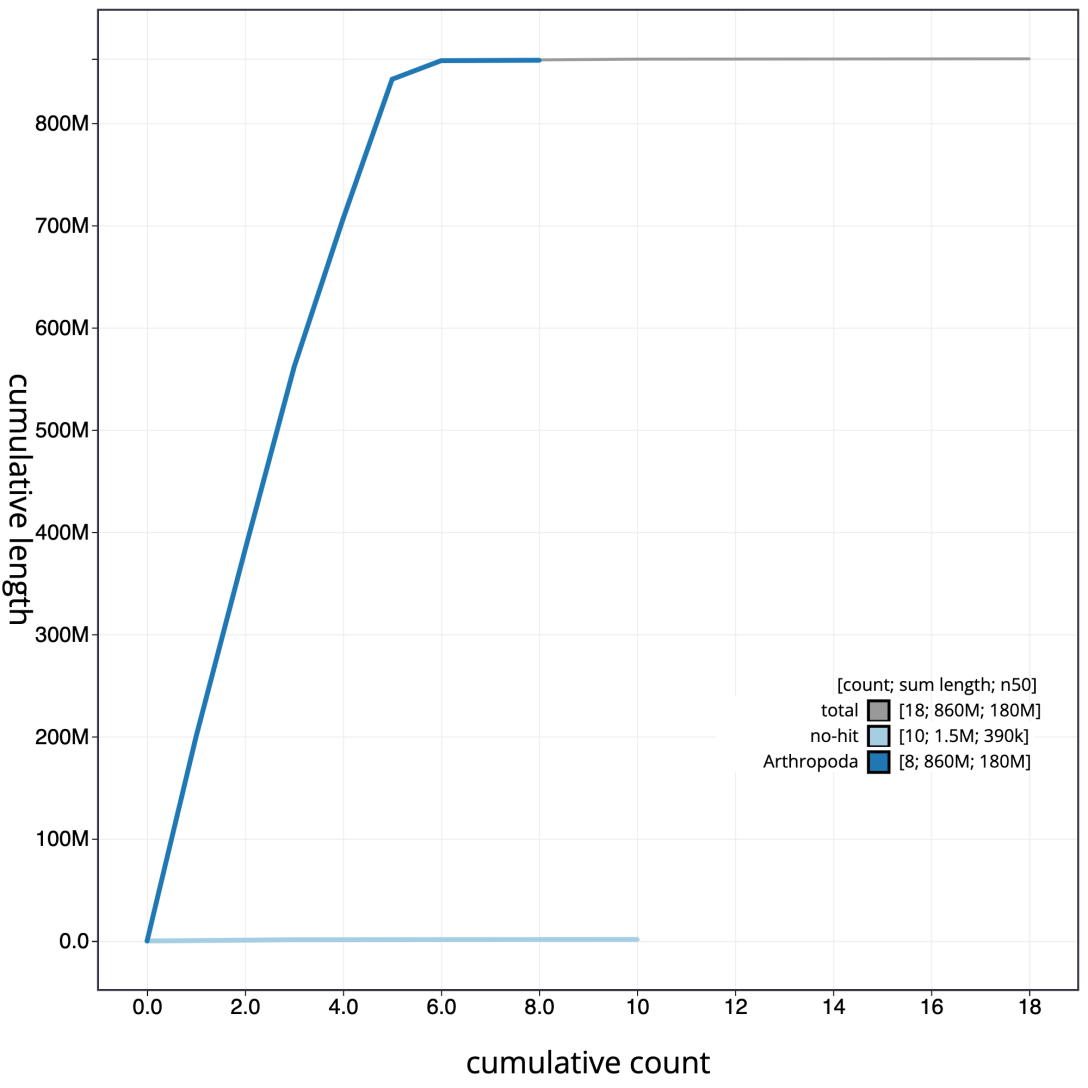
Genome assembly of *Pherbina coryleti*, idPheCory1.1: cumulative sequence. BlobToolKit cumulative sequence plot. The grey line shows cumulative length for all scaffolds. Coloured lines show cumulative lengths of scaffolds assigned to each phylum using the buscogenes taxrule. An interactive version of this figure is available at https://blobtoolkit.genomehubs.org/view/idPheCory1.1/dataset/CALSEO01/cumulative.

**Figure 5.**
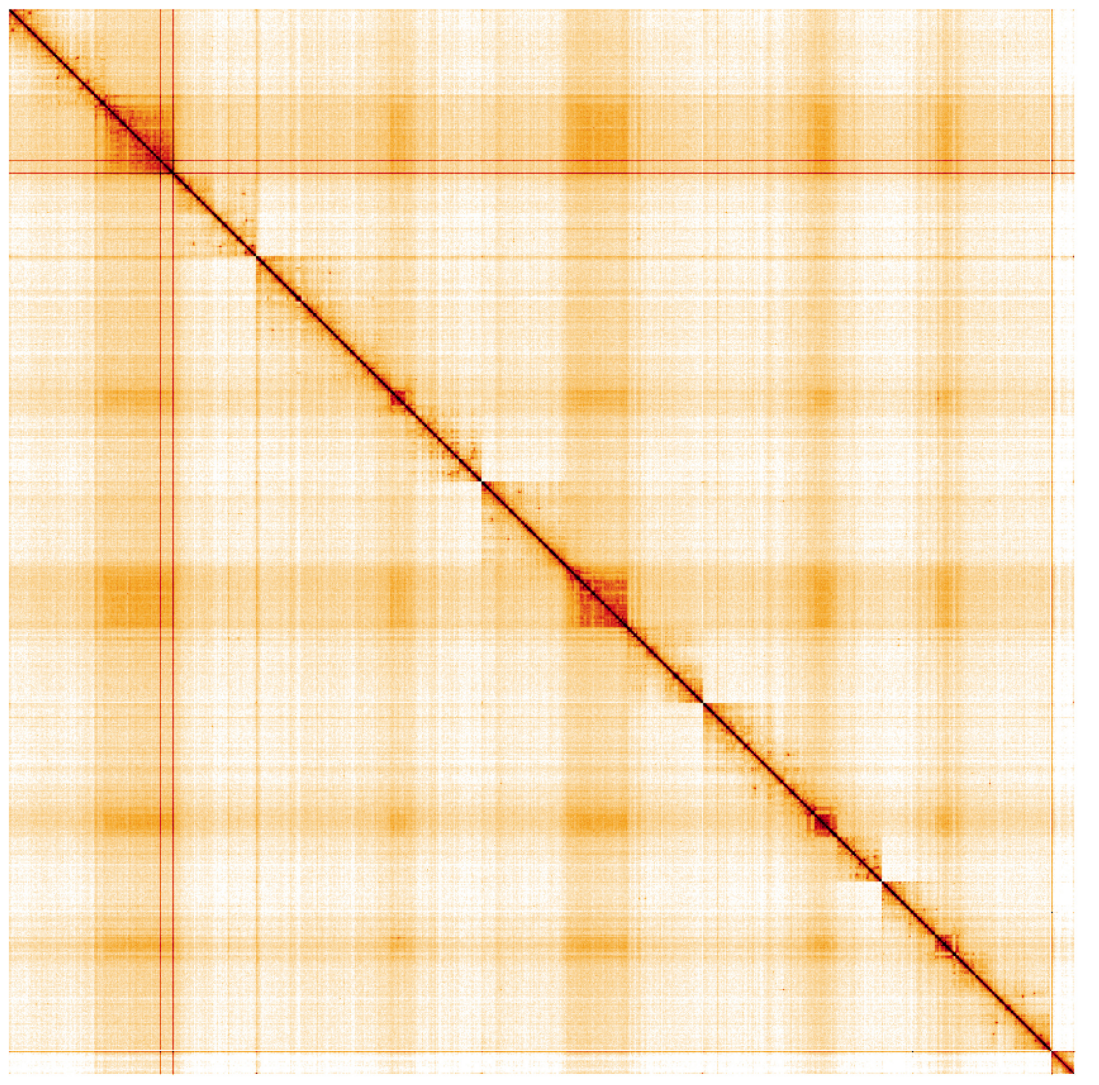
Genome assembly of *Pherbina coryleti*, idPheCory1.1: Hi-C contact map. Hi-C contact map of the idPheCory1.1 assembly, visualised using HiGlass. Chromosomes are shown in order of size from left to right and top to bottom. An interactive version of this figure may be viewed at https://genome-note-higlass.tol.sanger.ac.uk/l/?d=Z4Bj68n2TiuM4jGCb-bNNw.

**Table 2.** Chromosomal pseudomolecules in the genome assembly of *Pherbina coryleti*, idPheCory1.

INSDC accession	Chromosome	Size (Mb)	GC%
OX030948.1	1	199.73	33.8
OX030949.1	2	182.71	33.3
OX030950.1	3	178.93	33.7
OX030951.1	4	145.05	33.5
OX030952.1	5	136.52	33.3
OX030953.1	X	18.28	33.7
OX030954.1	MT	0.02	30.2

### Genome annotation report

The *P. coryleti* genome was annotated using the Ensembl rapid annotation pipeline ( Table 1; https://rapid.ensembl.org/Pherbina_coryleti_GCA_943735915.1/). The resulting annotation includes 33,571 transcribed mRNAs from 32,619 protein-coding genes.

## Methods

### Sample acquisition and nucleic acid extraction

A live male *P. coryleti* specimen (idPheCory1) was collected from vegetation at Parsonage Moor, SU459998, Abington, UK (latitude 51.69, longitude –1.33) on 19 June 2021, by Olga Sivell and Ryan Mitchell (Natural History Museum, London) using an insect net. It was identified by Duncan Sivell, Natural History Museum, London, following Rozkošný (1984), Rozkošný (1987) and Ball (2017). The specimen (NHMUK014036906, Figure 1) was snap-frozen on dry ice. The tissue samples taken from it were stored in a CoolRack prior to genome sequencing.

DNA was extracted at the Tree of Life laboratory, Wellcome Sanger Institute (WSI). The idPheCory1 sample was weighed and dissected on dry ice with tissue set aside for Hi-C sequencing. Whole organism tissue was disrupted using a Nippi Powermasher fitted with a BioMasher pestle. High molecular weight (HMW) DNA was extracted using the Qiagen MagAttract HMW DNA extraction kit. Low molecular weight DNA was removed from a 20 ng aliquot of extracted DNA using 0.8X AMpure XP purification kit prior to 10X Chromium sequencing; a minimum of 50 ng DNA was submitted for 10X sequencing. HMW DNA was sheared into an average fragment size of 12–20 kb in a Megaruptor 3 system with speed setting 30. Sheared DNA was purified by solid-phase reversible immobilisation using AMPure PB beads with a 1.8X ratio of beads to sample to remove the shorter fragments and concentrate the DNA sample. The concentration of the sheared and purified DNA was assessed using a Nanodrop spectrophotometer and Qubit Fluorometer and Qubit dsDNA High Sensitivity Assay kit. Fragment size distribution was evaluated by running the sample on the FemtoPulse system.

### Sequencing

Pacific Biosciences HiFi circular consensus and 10X Genomics read cloud DNA sequencing libraries were constructed according to the manufacturers’ instructions. DNA sequencing was performed by the Scientific Operations core at the WSI on a Pacific Biosciences SEQUEL II (HiFi) instrument. Hi-C data were also generated from tissue of idPheCory1 using the Arima v2 kit and sequenced on the Illumina NovaSeq 6000 instrument.

### Genome assembly

Assembly was carried out with Hifiasm ( Cheng *et al.*, 2021) and haplotypic duplication was identified and removed with purge_dups ( Guan *et al.*, 2020). The assembly was scaffolded with Hi-C data ( Rao *et al.*, 2014) using YaHS ( Zhou *et al*., 2022). The assembly was checked for contamination as described previously ( Howe *et al.*, 2021). Manual curation was performed using HiGlass ( Kerpedjiev *et al.*, 2018) and Pretext ( Harry, 2022). The mitochondrial genome was assembled using MitoHiFi ( Uliano-Silva *et al.*, 2021), which performed annotation using MitoFinder ( Allio *et al.*, 2020). The genome was analysed and BUSCO scores were generated within the BlobToolKit environment ( Challis *et al.*, 2020). Table 3 contains a list of all software tool versions used, where appropriate.

**Table 3.** Software tools and versions used.

Software tool	Version	Source
BlobToolKit	3.3.10	Challis *et al.*, 2020
Hifiasm	0.16.1-r375	Cheng *et al.*, 2021
HiGlass	1.11.6	Kerpedjiev *et al.*, 2018
MitoHiFi	2	Uliano-Silva *et al.*, 2021
PretextView	0.2	Harry, 2022
purge_dups	1.2.3	Guan *et al.*, 2020
YaHS	yahs-1.1.91eebc2	Zhou *et al*., 2022

### Genome annotation

The BRAKER2 pipeline ( Brůna *et al.*, 2021) was used in Ensembl to generate draft annotation for the *P. coryleti* assembly (GCA_943735915.1).

### Ethics/compliance issues

The materials that have contributed to this genome note have been supplied by a Darwin Tree of Life Partner. The submission of materials by a Darwin Tree of Life Partner is subject to the Darwin Tree of Life Project Sampling Code of Practice. By agreeing with and signing up to the Sampling Code of Practice, the Darwin Tree of Life Partner agrees they will meet the legal and ethical requirements and standards set out within this document in respect of all samples acquired for, and supplied to, the Darwin Tree of Life Project. Each transfer of samples is further undertaken according to a Research Collaboration Agreement or Material Transfer Agreement entered into by the Darwin Tree of Life Partner, Genome Research Limited (operating as the Wellcome Sanger Institute), and in some circumstances other Darwin Tree of Life collaborators.

## Data Availability

European Nucleotide Archive: *Pherbina coryleti.* Accession number PRJEB52655; https://identifiers.org/ena.embl/PRJEB52655 ( Wellcome Sanger Institute, 2022). The genome sequence is released openly for reuse. The *Pherbina coryleti* genome sequencing initiative is part of the Darwin Tree of Life (DToL) project. All raw sequence data and the assembly have been deposited in INSDC databases. Raw data and assembly accession identifiers are reported in Table 1.

## Author information

Members of the Natural History Museum Genome Acquisition Lab are listed here: https://doi.org/10.5281/zenodo.5746819.

Members of the Darwin Tree of Life Barcoding collective are listed here: https://doi.org/10.5281/zenodo.4893703.

Members of the Wellcome Sanger Institute Tree of Life programme are listed here: https://doi.org/10.5281/zenodo.4783585.

Members of Wellcome Sanger Institute Scientific Operations: DNA Pipelines collective are listed here: https://doi.org/10.5281/zenodo.4790455.

Members of the Tree of Life Core Informatics collective are listed here: https://doi.org/10.5281/zenodo.5013541.

Members of the Darwin Tree of Life Consortium are listed here: https://doi.org/10.5281/zenodo.4783558.
