# Supplementary material for: The genome sequence of a snail-killing fly, Pherbina coryleti (Scopoli, 1763)
Source: Wellcome Open Res. 2023 Jan 19;8:30. [Version 1] doi: 10.12688/wellcomeopenres.18790.1 (PMC10616662; doi:10.12688/wellcomeopenres.18790.1)
Supplement: Peer review history

# Reviewer report: Reviewer response for version 1

**DOI:** 10.21956/wellcomeopenres.20835.r67664

**Published:** 2023-10-30

**Authors:** Liping Yan

**Affiliations:** 1 Beijing Forestry University, Beijing, China

The manuscript is well prepared, with methodology detailly written, results clearly reported. It seems to me, the Hi-C contact map could be improved, and the sequence depth is not moderate but high enough when using DNA extracted from a single fly individual.

 The mitogenome of *Pherbina coryleti* is assembled, the access of which should also be documented.

Are sufficient details of methods and materials provided to allow replication by others?

Yes

Is the rationale for creating the dataset(s) clearly described?

Yes

Are the datasets clearly presented in a useable and accessible format?

Yes

Are the protocols appropriate and is the work technically sound?

Yes

Reviewer Expertise:

Phylogenomics of Diptera, genomics

I confirm that I have read this submission and believe that I have an appropriate level of expertise to confirm that it is of an acceptable scientific standard.

# Reviewer report: Reviewer response for version 1

**DOI:** 10.21956/wellcomeopenres.20835.r62301

**Published:** 2023-08-09

**Authors:** Sanjay Kumar Pradhan

**Affiliations:** 1 Department of Agricultural Entomology, College of Agriculture, University of Agricultural Sciences, Bengaluru, Karnataka, India

The manuscript is well structured, and the analysis covered is justifying the core research question raised. Some technical and typographical errors are notified for corrections in the PDF version (in track change mode for reference). Authors can look into the suggestions for further improvements. I approve the manuscript with some minor revisions.

Are sufficient details of methods and materials provided to allow replication by others?

Yes

Is the rationale for creating the dataset(s) clearly described?

Yes

Are the datasets clearly presented in a useable and accessible format?

Yes

Are the protocols appropriate and is the work technically sound?

Yes

Reviewer Expertise:

Viral genomics in insects, genetic engineering

I confirm that I have read this submission and believe that I have an appropriate level of expertise to confirm that it is of an acceptable scientific standard.
